# Electroencephalographic biomarkers of antibody-mediated autoimmune encephalitis

**DOI:** 10.3389/fneur.2025.1510722

**Published:** 2025-03-26

**Authors:** Lu Sun, Yaping Hu, Jingjing Yang, Lihong Chen, Ying Wang, Wei Liu, Jau-Shyong Hong, Yunhui Lv, Lin Yang, Ying Wang

**Affiliations:** ^1^Department of Neurology, The First Affiliated Hospital of DaLian Medical University, Da Lian, China; ^2^Neuropharmacology Section, Neurobiology Laboratory, National Institute of Environmental Health, Sciences, Research Triangle Park, Durham, NC, United States

**Keywords:** antibody-mediated autoimmune encephalitis, electroencephalography, anti-LGI1 encephalitis, anti-NMDAR encephalitis, anti-GABA_B_R encephalitis, anti-Caspr2 encephalitis, anti-GAD65 encephalitis, MOG antibody cortical encephalitis

## Abstract

**Objective:**

To identify electroencephalographic (EEG) biomarkers for different subtypes of antibody-mediated autoimmune encephalitis (AE) and assess their significance in disease severity, treatment response, and prognosis.

**Methods:**

The clinical and EEG data from 60 AE patients were analyzed. The relationship between EEG severity in the acute phase and disease severity, treatment response, and prognosis was examined to identify factors contributing to poor outcomes.

**Results:**

The cohort included 60 patients with the following subtypes of encephalitis: anti-LGI1 (22), anti-NMDAR (12), anti-GABA_B_R (7), anti-GAD65 (6), anti-MOG (7), anti-Caspr2 (4), and GFAP-A (2). EEG abnormalities were detected in 96.7% of patients, higher than imaging abnormalities (66.7%, *p* < 0.05). Common EEG features included focal (86.7%) or diffuse (13.3%) slow waves, interictal epileptiform discharges (IEDs) in temporal (46.7%) or extratemporal (15%) regions, and clinical or subclinical seizures (36.7%). During the recovery phase, 92.6% of 27 patients showed significant improvement in EEG patterns, with reduced slow waves and IEDs. Specific EEG patterns were associated with different antibody subtypes. Anti-LGI1 encephalitis had two clinical-electroencephalographic patterns: one was MTLE-like seizure with ictal activity originating from the temporal region; the other was FBDS with ictal EEG showing generalized electro-decremental activity before or at the onset of seizure with extensive infra-slow activity superimposed with EMG artifacts. Anti-NMDAR encephalitis was marked by abnormal background activity, including extreme delta brush, frontotemporal delta activity, diffuse or focal slow waves, with scattered and unfixed IEDs. MOG antibody cortical encephalitis usually presented as diffuse or focal slow waves in unilateral or bilateral hemisphere accompanied by ipsilateral IEDs, sometimes with periodic lateralized epileptiform discharges (PLEDs). Anti-GABA_B_R and anti-GAD65 encephalitis usually exhibited slow waves, IEDs and ictal activity involving the temporal regions. The EEG severity grading correlated positively with disease severity (*r* = 0.547, *p* < 0.0001) and prognosis score (*r* = 0.521, *p* < 0.0001). Further ROC curve and binary logistics regression analysis showed moderate to severe abnormal EEG was a risk factor for poor prognosis (OR = 11.942, *p* < 0.05), with an AUC of 0.756.

**Conclusion:**

EEG is a sensitive and valuable tool for AE and exhibit common and specific features across different AE subtypes. The severity of EEG abnormalities is a strong predictor of disease outcome.

## 1 Introduction

Autoimmune encephalitis (AE) is a group of central nervous system inflammatory diseases mediated by autoimmune mechanisms. According to different antigens targeted by the immune response, AE can be subdivided into the following types: intra-neuronal antibody-mediated encephalitis, neuronal surface antibody-mediated encephalitis, intraneuronal synaptic protein (e.g., glutamate decarboxylase, GAD) antibody-mediated encephalitis which is between the two types mentioned above, and other AE without a definite antigen.

The antibodies of intra-neuronal antibody-mediated encephalitis target intraneuronal antigens, mediating irreversible neuronal damage through cellular immune mechanisms, with poor response to immunotherapy and usually accompanied by tumors. Since the identification of anti-N-methyl-D-aspartate receptor (NMDAR) encephalitis in 2007 (1), several neuronal surface antibodies have been identified, including Leucine-rich glioma inactivated protein 1 (LGI1) (2), gamma-aminobutyric acid receptor B (GABA_B_R) (3), contactin-associated protein-like 2 (Caspr2) antibody (4), etc. These antibodies target antigens located on the neuronal surface, mediating relatively reversible neuronal dysfunction through humoral immune mechanisms, with good response to immunotherapy (5). Anti-GAD65 encephalitis is intermediate between the above two types, with antibodies targeting intraneuronal synaptic protein, and the response to immunotherapy and prognosis remain unclear. Myelin oligodendrocyte glycoprotein immunoglobulin G (MOG-IgG) associated disease (MOGAD) is an inflammatory demyelinating disease of the central nervous system mediated by MOG-IgG. As shown by previous studies, 20.7% of the patients with MOGAD presented with typical encephalitis symptoms (6). Additionally, 70.5% of patients with glial fibrillary acidic protein astrocytopathy (GFAP-A), a novel autoimmune neurological disease that was first reported in 2016 (7), presented as encephalitis or meningitis. Therefore, we refer to encephalitis mediated by these different antibodies as antibody-mediated AE. Most patients with antibody-mediated AE have epileptic seizures, and seizure types vary across different subtypes of AE. As shown by previous studies, anti-LGI1encephalitis usually presented 2 kinds of seizure types: facial-brachial dystonia-like seizure (FBDS) and medial temporal lobe epilepsy-like (MTLE-like)seizure; anti-NMDAR encephalitis had more generalized than focal seizures; and anti-GABA_B_R encephalitis was characterized by refractory seizures as initial symptom, mainly generalized tonic–clonic seizure (GTCS) or MTLE-like seizure (8).

The diagnosis of AE relies on multi-dimensional assessment, including clinical presentation, imaging, electroencephalography (EEG), detection of autoantibodies, and response to immunotherapy. Among them, video electroencephalography (vEEG) plays a crucial role. The diversity of autoantibodies makes the EEG changes relatively complex, with both similarities and specific features across different subtypes. Previous studies have shown that around 85% of AE patients exhibited EEG abnormalities, which were usually non-specific, including diffuse or focal slow waves, interictal epileptic discharges (IEDs) located in the temporal lobe or other brain regions, some patients showed extreme delta brush (EDB) or periodic lateralized epileptiform discharges (PLEDs), focal seizures and subclinical seizures were frequent (69.6%), originating from various regions including the frontal, temporal, central, and parietal regions (9, 10). However, most studies focused on one or two types of antibodies, with small sample size and inconsistent results (11–13). So far, it remains unclear whether there are EEG biomarkers that can predict disease severity, treatment response, and prognosis.

Therefore, this study analyzed the clinical and EEG data of 60 AE patients, to clarify the common and specific EEG features of different subtypes of AE, and thereby identify their EEG biomarkers; and to define the role of EEG changes on disease severity, treatment response, and prognosis, as well as the risk factors for poor prognosis.

## 2 Materials and methods

### 2.1 Patients

Sixty patients diagnosed with antibody-mediated AE at the First Affiliated Hospital of Dalian Medical University between January 2013 and October 2023 were included in this study. All patients met the diagnostic criteria outlined in the Chinese Expert Consensus on the Diagnosis and Management of Autoimmune Encephalitis (2022 edition) (5). Patients were excluded if they had a history of severe systemic disease, psychiatric disorders, other neurological diseases (e.g., cerebrovascular disease, spinal cord disease, intracranial infection, or tumor), a history of traumatic brain injury or neurosurgery, or if they or their families were unwilling to provide informed consent.

The study was approved by the Medical Ethics Committee of the First Affiliated Hospital of Dalian Medical University, and written informed consent was obtained from all participants or their legal representatives.

### 2.2 Methods

Clinical data were collected and analyzed, including demographic information, clinical presentations, seizure semiology, EEG, imaging, laboratory data, as well as treatment and outcomes. All patients underwent 1.5 T/3.0 T multimodal brain magnetic resonance imaging (MRI) scans, and antibodies in cerebrospinal fluid (CSF) and serum were detected using cell-based assays conducted by KingMed Diagnostic Company (Shenyang, China). EEG recordings (2 or 24-h vEEG) were performed using a 32-channel Nihon Kohden EEG-1200C electroencephalograph with the international 10–20 system for scalp electrode placement. EEG severity grading was classified into five categories based on background activity and paroxysmal abnormalities: normal, borderline, mild, moderate, severe, and extremely severe (Supplementary Table S1). The evaluation of EEG severity grading was conducted by two experienced electroencephalographers who were blinded to the patients’ conditions or modified Rankin Scale (mRS, Supplementary Table S2) scores. Disease severity during the acute phase and prognosis 6 months post-discharge were assessed using the modified Rankin Scale (mRS) by two clinicians. The standard Rankin scale lacks the specificity necessary for accurately evaluating seizure frequency and changes in cognitive function. Therefore, this scale was refined to place greater emphasis on assessing seizures and cognitive function, thereby providing a more objective and comprehensive evaluation of disease severity, treatment response, and prognosis in patients with autoimmune encephalitis. Patients with an mRS score < 2 were classified as having a good prognosis, while those with a score ≥ 2 were categorized as having a poor prognosis. Treatment response was evaluated by comparing mRS scores from the acute phase to follow-up. A reduction of ≥2 points indicated a significantly effective response, a 1-point reduction was deemed effective, and no reduction or an increase in mRS score indicated an ineffective response.

### 2.3 Statistical analysis

Statistical analysis was performed using SPSS 26.0, GraphPad Prism 9.0, Origin 2022, and MedCalc 20.217. Continuous variables (e.g., age of onset, disease duration) were presented as mean ± standard deviation or median, while categorical data (e.g., gender, EEG severity grading) were reported as frequencies or percentages. Group comparisons for normally distributed variables were performed using the t-test, and non-normally distributed variables were compared using the Kruskal-Wallis H test. Correlations were assessed using Spearman’s rank correlation. Binary logistic regression analysis was used to identify prognostic factors, and receiver operating characteristic (ROC) curves were used to evaluate the predictive value of each indicator. A *p*-value < 0.05 was considered statistically significant.

## 3 Results

### 3.1 General information

Between January 2013 and October 2023, 60 patients with antibody-mediated AE were enrolled, including 22 with anti-LGI1 encephalitis, 12 with anti-NMDAR encephalitis, 7 with anti-GABA_B_R encephalitis, 4 with anti-Caspr2 encephalitis, 6 with anti-GAD65 encephalitis, 7 with MOG-IgG associated cortical encephalitis (FLAMES), and 2 with GFAP-A encephalitis. The cohort comprised 30 males and 30 females, with a sex ratio of 1:1. The mean age at onset was 47.35 ± 19.25 years (range: 14–83), and the median disease duration was 30 days. MRI abnormalities were observed in 40 patients (66.7%). Supplementary Figure S1 showed the sex distribution of different types of AE.

### 3.2 EEG features of antibody-mediated AE

#### 3.2.1 Common EEG features

All 60 patients underwent 2 or 24-h vEEG during the acute phase, with abnormalities detected in 96.7% (58/60), higher than those detected by imaging (66.7%, 40/60), *p* < 0.05. Background activity abnormalities included focal slow waves in 86.7% (52/60), diffuse slow waves in 10% (6/60), and unilateral diffuse slow waves in 5% (3/60) of patients. Slow waves were located in unilateral hemisphere in 25% (15/60), and in bilateral hemispheres in 71.7% (43/60) of patients; 30% (18/60) showed delta activity or rhythm, and only 1 patient with anti-NMDAR encephalitis showed extreme delta brush (EDB). Interictal epileptiform discharges (IEDs) were found in 61.7% (37/60) of patients, predominantly in the temporal region (46.7%, 28/60). Clinical seizures were recorded in 31.7% (19/60), with subclinical seizures in 5% (3/60)of patients. Supplementary Figure S2 showed distribution map of abnormal EEG findings across different subtypes of antibody-mediated encephalitis.

During the recovery phase, 27 of 60 patients underwent long-term vEEG, and 92.6% showed significant improvement in EEG patterns, with reduced slow waves and IEDs, and no ictal activity was observed. Note the recovery phase referred to 10 days to 6 months or even longer after immunotherapy, distinct from spontaneous recovery.

#### 3.2.2 Specific EEG features by subtype

##### 3.2.2.1 Anti-LGI1 encephalitis

This group included 22 patients (10 males and 12 females) with a sex ratio of 10:12. The average onset age was 61 ± 13 years (range 21–83), with a median disease duration of 75 days. EEG abnormalities were present in all cases. Based on clinical semiology, the patients were subdivided into 3 groups: MTLE-like seizure group (12 cases), FBDS group (9 cases), and MTLE-like seizure + FBDS group (1 case).

**Figure 1:**
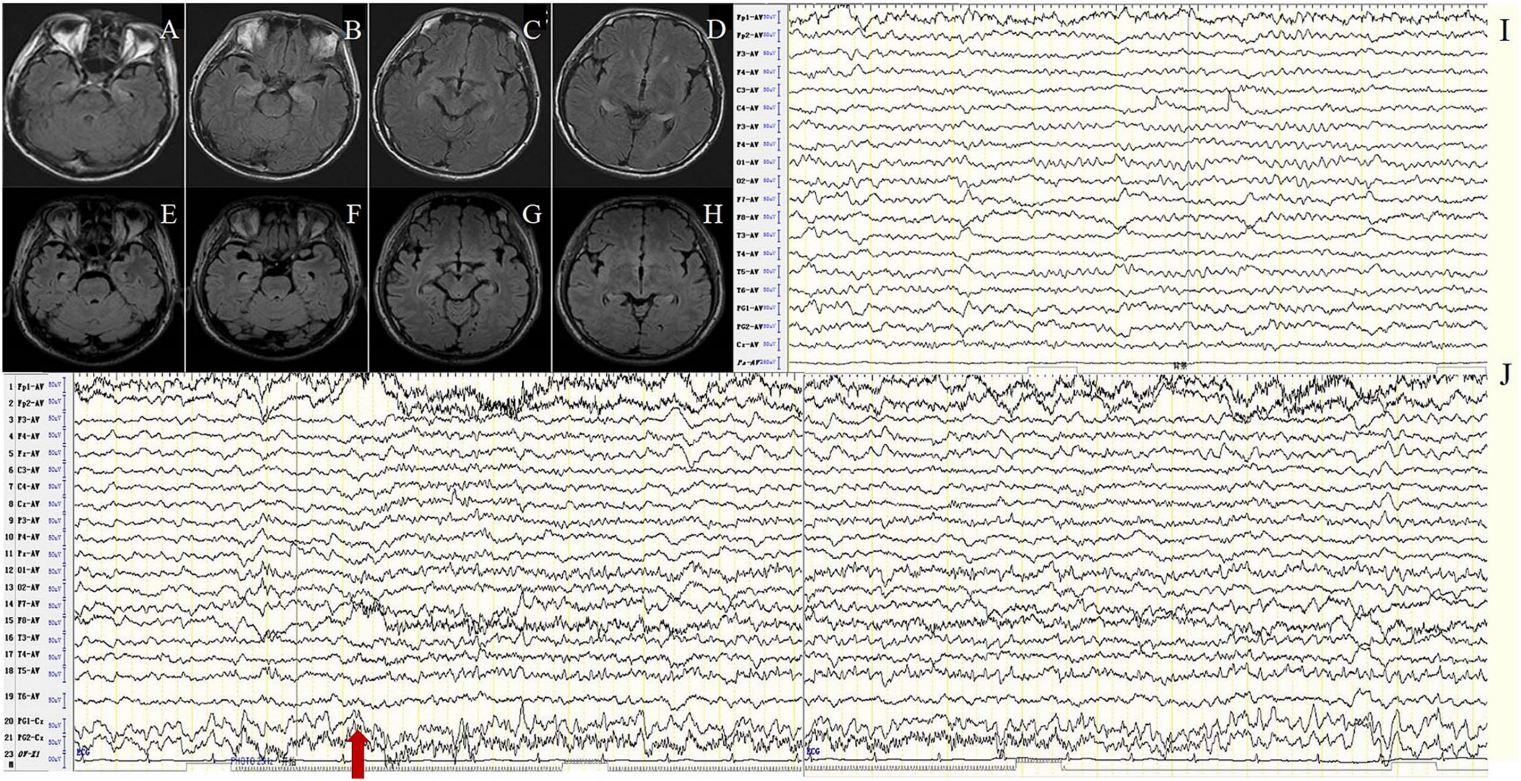
Case 7, male, 62 years old, who was diagnosed with anti-LGI1encephalitis, Mesial temporal lobe epilepsy (MTLE)-like seizure group. **(A–D)** Twenty-three days after onset and before immunotherapy, brain MRI showed T2 FLAIR hyperintensity on the bilateral hippocampus; **(E–H)** Two months after immunotherapy, brain MRI showed significant improvement in the abnormalities. **(I–J)** Before immunotherapy, EEG showed slow waves in the background activity in the bilateral frontotemporal regions **(I)**, and subclinical electroencephalographic seizure originating from bilateral temporal regions **(J)**. The red arrow indicates the beginning of the subclinical electroencephalographic seizure.

In the MTLE-like seizure group, the background activity was abnormal in all 12 patients (100%), including focal slow waves in 10 (83.3%) and diffuse slow waves in 2 patients (16.7%). IEDs were detected in the unilateral or bilateral temporal regions in 8 patients (66.7%). MTLE-like seizures were detected in 4 patients (33.3%), with the ictal EEG showing unilateral or bilateral temporal region origin (Figure 1), and subclinical seizures originating from bilateral temporal regions were detected in 2 patients (16.7%).

**Figure 2:**
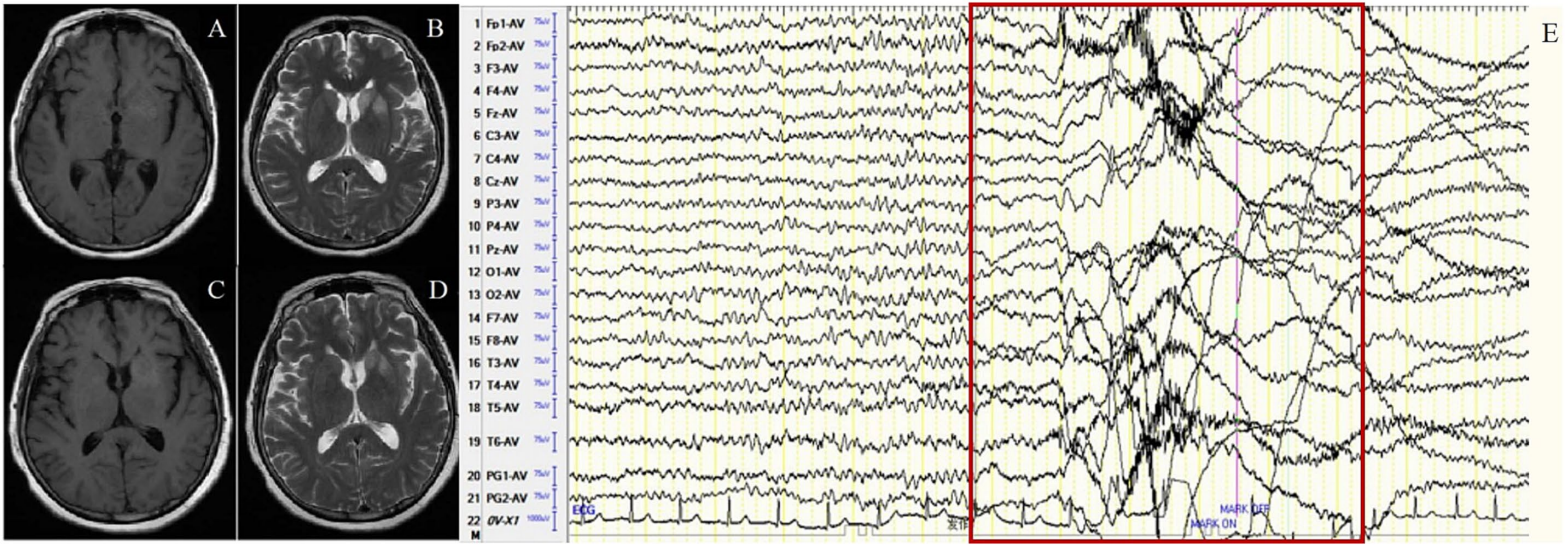
Case 13, female, 63 years old, who was diagnosed with anti-LGI1encephalitis, Faciobrachial dystonic seizure (FBDS) group. **(A,B)** Nineteen days after onset and before immunotherapy, brain MRI showed T1 and T2 hyperintensity on the left basal ganglia. **(C,D)** Ten days after immunotherapy, brain MRI showed significant improvement in the abnormalities on the left basal ganglia. **(E)** Before immunotherapy, ictal EEG of FBDS showed generalized electro-decremental activity 1 s before the onset of seizure with extensive medium-high amplitude infra-slow activity superimposed with a large number of EMG artifacts. The red border signifies the ictal EEG of FBDS.

In the FBDS group, the background activity was abnormal in 8 of 9 patients (88.9%), presenting as focal slow waves in the frontal, central, and temporal regions. IEDs were detected in 4 patients (44.4%) with unfixed location, among them 2 in the temporal region and 2 in the frontocentral region. A total of 73 times of FBDS were detected in 9 cases, with ictal EEG showing generalized electro-decremental activity before or at the onset of the seizure with extensive medium-high amplitude infra-slow activity superimposed with a large number of EMG artifacts (Figure 2). Further analysis of 73 times of FBDS revealed that 67 showed electro-decremental activity before the seizures (0.6 s to 4.9 s before the seizures) and 6 showed electro-decremental activity during the seizures; all 73 showed widespread medium-to-high-amplitude, multi-phasic slow waves, with amplitude ranging from 68.4 μV to 1,000 μV, frequency ranging from 0.2 Hz to 0.7 Hz, and durations ranging from 0.8 s to 2.8 s; EMG was simultaneously recorded in 44 seizures, and the EEG changes occurred 0 s to 2.8 s before the EMG changes.

There was only 1 patient in the MTLE-like seizure + FBDS group, who initially presented with MTLE-like seizures and developed FBDS during treatment, and both types of ictal EEG were present.

Ten of the 22 patients underwent 2 or 24-h vEEG 6 months after immunotherapy. One patient showed no improvement, while other patients showed significant improvement with reduced slow waves and IEDs, and no ictal activity was detected. The clinical and EEG characteristics of anti-LGI1 encephalitis were shown in Table 1.

**Table 1:** Clinical and electroencephalographic characteristics of anti-Leucine-rich Glioma Inactivated 1 (LGI1) Encephalitis.

Case	Sex	Age	Clinical manifestations	Brain MRI	vEEG	LGI1 Ab titer in CSF/serum	Disease severity (mRS)	Treatment	Therapeutic response	Outcome (mRS)
1	F	46	MTLE-like seizures, cognitive dysfunction, auditory hallucinations	Normal	Background: focal slow waves in bilateral temporal regions	1:10/1:1000	3	Corticosteroids+ IVIG+LEV	Significantly effective	1
2	M	50	MTLE-like seizures, cognitive dysfunction, anxiety and depression, hyponatremia	Normal	Background: focal slow waves in bilateral frontal, central and temporal regions; Interictal: sharp and wave complexes in bilateral frontotemporal regions; Itcal:3 MTLE-like seizures	1:10/1:100	3	Corticosteroids+ LEV	Significantly effective	0
3	M	58	MTLE-like seizures, cognitive dysfunction, hyponatremia	T2 FLAIR hyperintensity in left hippocampus	Background: diffuse slow waves	1:32/1:320	4	Corticosteroids+ LEV	Significantly effective	2
4	M	59	MTLE-like seizures, cognitive dysfunction, anxiety and depression, hyponatremia	T2 FLAIR hyperintensity in right hippocampus	Background: focal slow waves in right occipital, parietal and posterior temporal regions	1:3.2/1:100	4	Corticosteroids+ IVIG+VPA	Significantly effective	1
5	F	40	MTLE-like seizures, cognitive dysfunction, anxiety and depression	Atrophy and T2 FLAIR hyperintensity in right hippocampus, decreased NAA peak in bilateral hippocampus	Background: focal slow waves in bilateral frontal, central and temporal regions; Interictal: sharps in right temporal region; Itcal:4 MTLE-like seizures and 3 subclinical seizures	−/1:10	3	Corticosteroids+ IVIG+OXC	Effective	0
6	F	56	MTLE-like seizures, anxiety and depression, sleep disorders, hyponatremia	T2 FLAIR hyperintensity in bilateral hippocampus	Background: focal slow waves in bilateral frontal, temporal and central regions; Interictal: sharps in bilateral temporal regions; Itcal:4 MTLE-like seizures	1:1/1:10	4	Corticosteroids+ IVIG+LEV	Significantly effective	2
7	M	62	MTLE-like seizures, cognitive dysfunction, mental and behavioral abnormalities, hyponatremia	Swelling and T2 FLAIR hyperintensity in bilateral hippocampus and amygdala	Background: focal slow waves in frontotemporal regions; Interictal: sharp and wave complexes in bilateral temporal regions; Ictal:12 subclinical seizures	1:1/1:32	4	Corticosteroids+ IVIG+LEV	Significantly effective	2
8	M	74	MTLE-like seizures, cognitive dysfunction, mental and behavioral abnormalities, hyponatremia	Swelling and T2 FLAIR hyperintensity in right hippocampus	Background: diffuse slow waves, generalized delta activity	1:32/1:32	3	Corticosteroids+ LEV	Significantly effective	1
9	M	56	MTLE-like seizures, cognitive dysfunction	Swelling and T2 FLAIR hyperintensity in left hippocampus	Background: focal slow waves in bilateral frontotemporal regions; Interictal: sharps in left anterior temporal region	1:32/1:100	3	Corticosteroids+ IVIG+OXC	Significantly effective	1
10	M	61	MTLE-like seizures, cognitive dysfunction, anxiety and depression, hallucinations	Atrophy and T2 FLAIR hyperintensity in left hippocampal	Background: focal slow waves in bilateral frontal, central and temporal regions	−/1:32	3	Corticosteroids+ LEV	Significantly effective	2
11	M	45	MTLE-like seizures, cognitive dysfunction, mental and behavioral abnormalities, hyponatremia	T2 FLAIR hyperintensity in right hippocampus and amygdala, decreased NAA peaks in bilateral hippocampus	Background: focal slow waves in bilateral temporal regions; Interictal: sharps in right anterior temporal region	1:10/1:100	3	Corticosteroids+ LEV	Significantly effective	1
12	M	68	MTLE-like seizures, cognitive dysfunction, depression	^18^FDG PET-CT: enhanced FDG metabolism in right caudate nucleus	Background: focal slow waves in bilateral frontal, central, and temporal regions	1:10/1:10	4	Corticosteroids+ IVIG +LEV+ PA + LCM	Significantly effective	0
13	F	63	FBDS, cognitive dysfunction, anxiety and depression, increased sleep, hyponatremia	T2 FLAIR hyperintensity in left basal ganglia	Background: focal slow waves in bilateral frontal and central regions; Interictal: sharps in bilateral frontal and central regions	1:10/1:100	5	Corticosteroids+ IVIG+ ASMs	Ineffective	6
14	F	21	FBDS, cognitive dysfunction, anxiety and depression, hyponatremia	T2 FLAIR hyperintensity in right caudate nucleus	Background: focal slow waves in bilateral frontal, central and temporal regions	1:3.2/1:100	3	CBZ	Significantly effective	1
15	F	65	FBDS, cognitive dysfunction, mental and behavioral abnormalities	Normal	Background: focal slow waves in bilateral frontal, central and temporal regions; Ictal: more than 20 FBDS	1:32/1:10	3	Corticosteroids+ IVIG+OXC	Significantly effective	2
16	F	57	FBDS, cognitive dysfunction, personality changes, hallucinations	Normal	Background: focal slow waves in bilateral frontal, central and temporal regions; Interictal: sharps in bilateral temporal regions	1:100/1:100	3	Corticosteroids+ IVIG+CBZ	Significantly effective	1
17	F	67	FBDS, cognitive dysfunction	^18^F-FDG PET-CT: enhanced FDG metabolism in bilateral lentiform nuclei	Background: focal slow waves in bilateral frontal, parietal and temporal regions; Ictal:20 FBDS	−/1:32	4	Corticosteroids+ IVIG+VPA	Effective	2
18	F	68	FBDS, cognitive dysfunction, anxiety and depression, hyponatremia	Normal	Background: focal slow waves in bilateral frontal, parietal and temporal regions; interictal: sharps in bilateral frontal pole, frontal, and midline regions	1:10/1:10	2	Corticosteroids+ IVIG+LEV	Significantly effective	0
19	F	61	FBDS, cognitive dysfunction, anxiety and depression	Normal	Background: focal slow waves in bilateral frontotemporal regions; Ictal: 30 FBDS	1:32/1:32	4	Corticosteroids+ IVIG+ LEV+CBZ	Significantly effective	0
20	M	70	FBDS, cognitive dysfunction	Normal	Background: focal slow waves in bilateral frontal and central regions; Ictal: dozens of FBDS	1:100/1:320	4	Corticosteroids+ IVIG+LEV +CBZ + VPA + Mycophenolate mofetil	Significantly effective	2
21	F	83	FBDS, cognitive dysfunction, anxiety and depression	Normal	Background: focal slow waves in bilateral frontotemporal regions; Interictal: spikes, sharp and wave complexes in left temporal region; Ictal: 16 FBDS	1:32/1:100	3	IVIG+CBZ + LCM + Mycophenolate mofetil	Effective	2
22	F	67	MTLE-like seizures, FBDS, cognitive dysfunction, anxiety and depression	T2 FLAIR hyperintensity in bilateral hippocampus	Background: focal slow waves in bilateral temporal region; Interictal: sharp and wave complexes in bilateral temporal regions	1:10/1:100	3	Corticosteroids+ IVIG+OXC	Effective	2

ASMs, anti-seizure medication; CBZ, carbamazepine; CSF, cerebrospinal fluid; FBDS, faciobranchial dystonia seizure; IVIG, intravenous immunoglobulin; LCM, lacoxamide; LEV, levetiracetam; MRI, magnetic resonance imaging; MTLE, mesial temporal lobe epilepsy; OXC, oxcarbazepine; PET-CT, positron emission tomography computed tomography; T2 FlAIR, T2 fluid attenuated inversion recovery; VPA, Valproate; 18F-FDG, 18F-flurodeoxyglucose; mRS, Modified Rankin Scale; NAA, N-acetyl aspartate.

##### 3.2.2.2 Anti-NMDAR encephalitis

**Figure 3:**
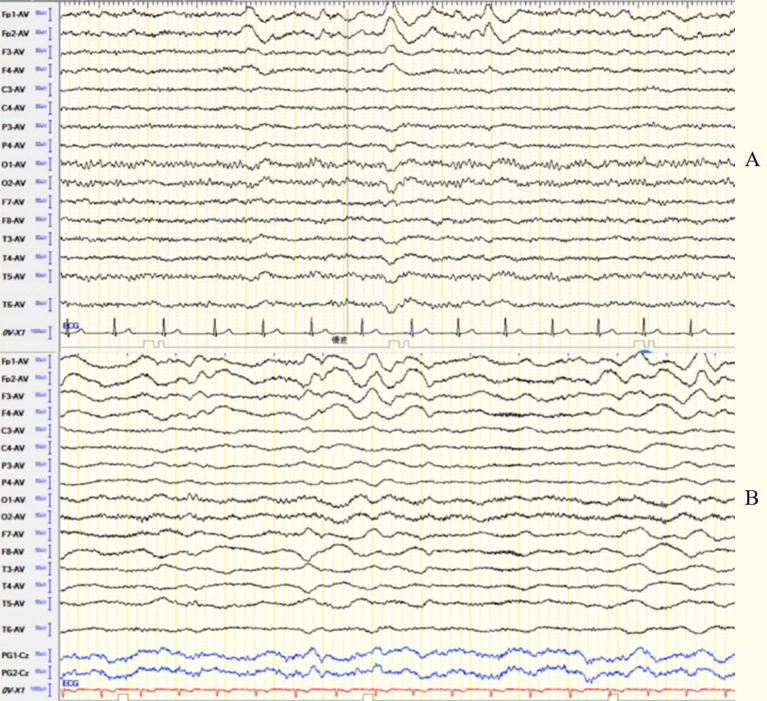
**(A)** Case 24, female, 27 years old, who was diagnosed with anti-NMDAR encephalitis. Before immunotherapy, EEG showed bilateral frontal delta activity or rhythm; **(B)** Case 26, male, 25 years old, who was diagnosed with anti-NMDAR encephalitis. Before immunotherapy, EEG showed generalized delta activity or rhythm.

This group consisted of 12 patients (6 males and 6 females) with a sex ratio of 1:1. The average onset age was 31.5 ± 13.47 years (range 14–63). The median disease duration was 30 days. EEG abnormalities were present in 91.7% of patients. The background activity was abnormal in 11 patients (91.7%), among them 1 showed EDB, 6 showed frontotemporal delta activity or rhythm (Figure 3), 3 showed bilateral focal slow waves, and 1 showed unilateral diffuse slow waves. IEDs were detected in 7 patients (58.3%) with scattered and unfixed location, including unilateral frontal pole and anterior temporal region in 2 patients, unilateral anterior and middle temporal region in 2 patients, unilateral posterior temporal and occipital region in 1 patient, unilateral anterior temporal and bilateral occipital regions in 1 patient, and periodic lateralized epileptiform discharges (PLEDs) in 1 patient. No ictal EEG was detected.

Six of the 12 patients underwent 2 or 24-h vEEG 2 to 18 months after immunotherapy, and all showed significant improvement with reduced slow waves, IEDs were still visible in 2 patients, and no ictal EEG was detected. The clinical and EEG characteristics of anti-NMDAR encephalitis were shown in Table 2.

**Table 2:** Clinical and electroencephalographic characteristics of Anti-N-methyl-D-aspartate Receptor (NMDAR) Encephalitis.

Case	Sex	Age	Clinical manifestations	Brain MRI	vEEG	NMDAR Ab titer in CSF/serum	Disease severity (mRS)	Treatment	Therapeutic response	Outcome (mRS)
23	F	24	Seizures, cognitive dysfunction, anxiety and depression	Normal	Background: delta activity or rhythm in bilateral frontotemporal regions	1:10/1:320	5	Corticosteroids+LEV +TPM+VPA	Significantly effective	2
24	F	27	Seizures, cognitive dysfunction, mental and behavioral abnormalities, sleep disorder	Scattered patchy T2 FLAIR hyperintensity in cortex and subcortex	Background: focal slow waves in bilateral frontotemporal regions; Interictal: sharp and wave complexes in right anterior temporal region	1:10/1:100	4	Corticosteroids+ VPA+LEV	Significantly effective	2
25	F	33	Seizures, cognitive dysfunction, anxiety and depression	Patchy T2 FLAIR hyperintensity in bilateral paraventricular region	Background: focal slow waves in left temporal region; Interictal: sharps in left posterior temporal region	1:3.2/1:10	3	Corticosteroids+ IVIG+CBZ	Significantly effective	0
26	M	25	Seizures, cognitive dysfunction, mental and behavioral abnormalities	Normal	Background: delta activity or rhythm in bilateral frontotemporal regions	1:3.2/1:10	5	Corticosteroids+ IVIG+LEV	Significantly effective	2
27	M	36	Cognitive dysfunction, mental and behavioral abnormalities	Patchy T2 FLAIR hyperintensity in subcortical white matter of left temporal lobe	Background: focal slow waves in bilateral frontal, central and temporal regions	1:1/−	2	Corticosteroids	Significantly effective	0
28	M	63	Dizziness, incomplete motor aphasia, ataxia, cognitive dysfunction	Normal	Background: theta activity or rhythm in bilateral frontal, central and temporal regions	−/1:32	3	Corticosteroids+ IVIG	Significantly effective	1
29	M	34	Cognitive dysfunction, mental and behavioral abnormalities	Normal	Background: focal slow waves in bilateral frontal, central and temporal regions	1:32/−	4	Corticosteroids	Effective	2
30	F	20	Seizures, cognitive dysfunction, mental and behavioral abnormalities	Normal	Background: extreme delta brush	1:10/1:32	5	Corticosteroids+ LEV	Effective	2
31	M	47	Ataxia, cognitive dysfunction, mental and behavioral abnormalities	Multiple patchy T2 FLAIR mild hyperintensities in bilateral frontotemporal-parietal regions, thalamus, and brainstem	Background: delta activity or rhythm in bilateral frontotemporal regions; Interictal: sharps in right temporal region	1:3.2/1:3.2	3	Corticosteroids+IVIG	Effective	1
32	M	36	Seizures, cognitive dysfunction, mental and behavioral abnormalities	Patchy T2 FLAIR hyperintensity in right occipitotemporal gyrus and hippocampus	Background: delta activity in right frontotemporal regions; Interictal: sharp and wave complexes in right anterior temporal region	1:32/1:32	3	Corticosteroids+VPA	Significantly effective	1
33	F	14	Seizures, headache, mental and behavioral abnormalities	Cortical swelling and T2 FLAIR hyperintensity in left frontal and insular cortex, decreased NAA peak and increased Cho peak	Background: diffuse slow waves in left hemisphere; Interictal: periodic epileptic discharges in left frontal region	1:1+/1:32+	5	Corticosteroids+IVIG+ LEV+VPA+Rituximab	Significantly effective	2
34	F	19	Seizures, cognitive dysfunction, mental and behavioral abnormalities	T2 FLAIR hyperintensity in left thalamus and right frontal and parietal regions	Background: delta activity in right anterior temporal region; Interictal: spikes in left anterior temporal region and bilateral occipital regions	1:10/1:10	4	Corticosteroids+ IVIG+LEV	Significantly effective	0

CBZ, carbamazepine; CSF, cerebrospinal fluid; IVIG, intravenous immunoglobulin; LEV, levetiracetam; MRI, magnetic resonance imaging; OXC, oxcarbazepine; T2 FlAIR, T2 fluid attenuated inversion recovery; TPM, topiramate; VPA, Valproate; mRS, Modified Rankin Scale; NAA, N-acetyl aspartate; Cho, choline.

##### 3.2.2.3 Anti-GABA_B_R encephalitis

**Figure 4:**
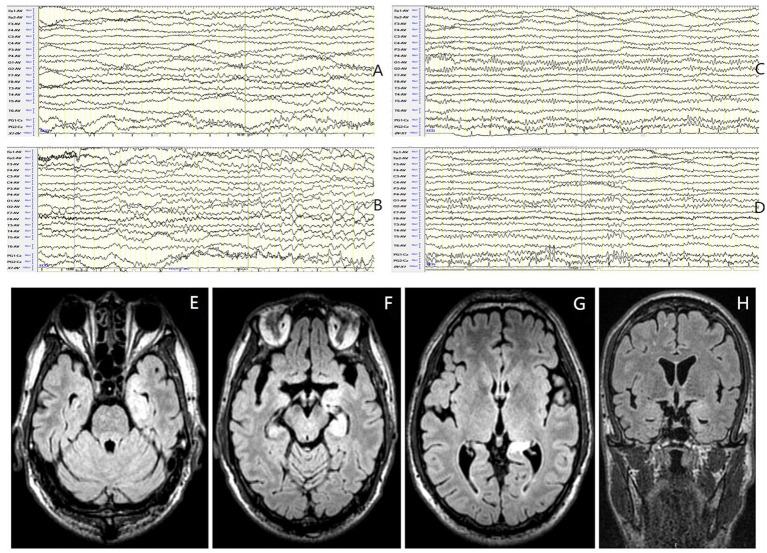
Case 39, male, 64 years old, who was diagnosed with anti-GABA_B_R encephalitis. **(A,B)** Twenty-one days after onset and before immunotherapy, EEG showed slow waves involving left temporal region in the background activity **(A)** and subclinical electroencephalographic seizure originating from left temporal region **(B)**; **(C,D)** Ten days after immunotherapy, the background activity improved significantly **(C)** with slow waves in the left posterior region **(D)**. **(E–H)** Before immunotherapy, brain MRI showed swelling of left hippocampus and T2 FLAIR hyperintensity in the bilateral hippocampus.

This group included 7 patients (6 males and 1 female) with a sex ratio of 6:1. The average onset age was 65 ± 8.9 years (range 46–73). The median disease duration was 14 days. EEG abnormalities were present in all patients (100%). The background activity was abnormal in 100% of cases, presenting as focal slow waves mainly involving the temporal regions (71.4%), and sometimes the frontal and central regions (57.1%), and slowing of the occipital α rhythm was detected in 2 patients (28.6%). IEDs were observed in 5 patients (71.4%) mainly involving the temporal region. and subclinical electrographic seizures originating from the left temporal region were detected in 1 patient (14.3%) (Figure 4).

Three of the 7 patients underwent 24-h vEEG 10 days to 8 months after immunotherapy, and all showed significant improvement with reduced slow waves, IEDs were still visible in 1 patient, and no ictal EEG was detected. The clinical and EEG characteristics of anti-GABA_B_R encephalitis are shown in Table 3.

**Table 3:** Clinical and electroencephalographic characteristics of Anti-Gamma-aminobutyric Acid B Receptor (GABA_B_R) Encephalitis.

Case	Sex	Age	Clinical manifestations	Brain MRI	vEEG	GABA_B_R Ab titer in CSF/serum	Disease severity (mRS)	Treatment	Therapeutic response	Outcome (mRS)
35	M	69	Seizures, cognitive dysfunction, mental and behavioral abnormalities	Swelling of left hippocampus, T2 FLAIR hyperintensity in bilateral hippocampus	Background: focal slow waves in bilateral temporal regions	1:1/1:1	5	Corticosteroids+ IVIG+VPA	Significantly effective	6
36	M	46	Seizures, anxiety	Decreased CBF within right temporal region	Background: focal slow waves in bilateral frontal, central and temporal regions; Interictal: sharps in left temporal region	1:10 /1:1000	2	Corticosteroids+ IVIG+CBZ	Effective	0
37	M	69	Seizures, cognitive dysfunction, anxiety and depression	^18^F-FDG PET-CT: decreased FDG metabolism in right temporal lobe	Background: focal slow waves in bilateral frontal, central and temporal regions; Interictal: sharp and wave complexes in right temporal region	1:10 /1:10	2	Corticosteroids+ IVIG+VPA	Significantly effective	0
38	F	69	Seizures, cognitive dysfunction, increased sleep	Normal	Background: focal slow waves in bilateral frontal and central regions; Interictal: sharps in right occipital region	1:32 /1:100	4	Corticosteroids+ LEV	Significantly effective	2
39	M	64	Seizures, cognitive dysfunction, mental and behavioral abnormalities	Normal	Background: focal slow waves in bilateral temporal regions; Interictal: sharps in left temporal region	1:32 /1:32	3	Corticosteroids+ CBZ	Ineffective	1
40	M	65	Seizures, cognitive dysfunction	Swelling of right hippocampus	Background: focal slow waves in right temporal region; Interictal: spikes or sharps in right anterior temporal regions	1:10/1:10	2	OXC	Effective	1
41	M	73	Seizures, cognitive dysfunction, mental and behavioral abnormalities	Normal	Background: focal slow waves in bilateral frontotemporal regions	1:100/1:100	5	Corticosteroids+IVIG+ Mycophenolate mofetil +VPA+LEV	Ineffective	6

CBZ, carbamazepine; CSF, cerebrospinal fluid; IVIG, intravenous immunoglobulin; LEV, levetiracetam; MRI, magnetic resonance imaging; OXC, oxcarbazepine; PET-CT, positron emission tomography computedtomography; T2 FlAIR, T2 fluid attenuated inversion recovery; VPA, Valproate; 18F-FDG, 18F-flurodeoxyglucose; mRS, Modified Rankin Scale.

##### 3.2.2.4 Anti-Caspr2 encephalitis

**Figure 5:**
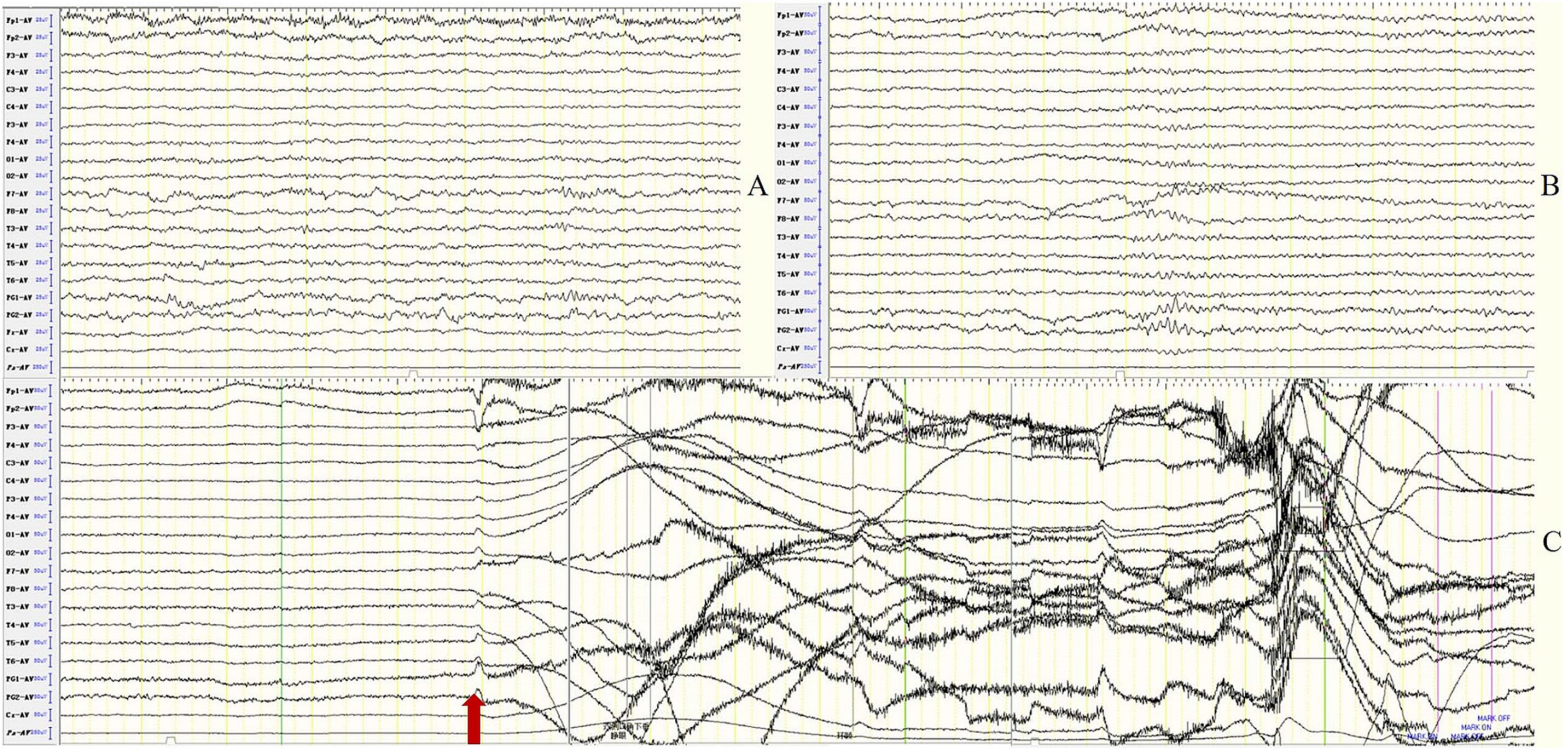
Case 43, male, 64 years old, who was diagnosed with anti-Caspr2 encephalitis. Ten days after onset and before immunotherapy, EEG showed focal slow waves in bilateral frontotemporal regions in the background activity **(A)** and atypical sharps in bilateral sphenoid electrodes **(B)**; **(C)** ictal EEG showed focal seizures originating from the right frontal region. The red arrow indicates the beginning of the seizure.

This group included 4 patients (2 males and 2 females) with a sex ratio of 1:1. The average onset age was 47.75 ± 12.9 years (range 35–64). The median disease duration was 365 days. EEG abnormalities were present in 75% of patients. The background activity was abnormal in 3 patients (75%) showing focal slow waves in bilateral frontal, central, and temporal regions. IEDs were detected in 2 patients (50%) involving the unilateral frontal pole and anterior temporal regions. Ictal EEG was detected in 2 patients, among them 1 showed 12 focal seizures originating from the right frontal region (Figure 5), while the other had 4 focal seizures with the ictal EEG showing generalized electro-decrement for 1–2 s.

Two of the 4 patients underwent 2 or 24-h vEEG 12 and 18 months after immunotherapy, respectively. One showed significant improvement in the background activity, but still had IEDs; no significant changes were observed in the other patient. The clinical and EEG characteristics of anti-Caspr2 encephalitis were shown in Table 4.

**Table 4:** Clinical and electroencephalographic characteristics of Anti-Contactin-associated Protein-2 (Caspr2) Encephalitis.

Case	Sex	Age	Clinical manifestations	Brain MRI	vEEG	Caspr2 Ab titer in CSF/serum	Disease severity (mRS)	Treatment	Therapeutic response	Outcome (mRS)
42	F	40	Seizures, cognitive dysfunction, anxiety and depression	Atrophy and T2 FLAIR hyperintensity in right hippocampus, decreased NAA peak in bilateral hippocampus	Background: focal slow waves in bilateral frontal, central and temporal regions; Interictal: sharps in right temporal region; Itcal: 4 MTLE-like seizures	−/1:10	3	Corticosteroids+I VIG+OXC	Effective	2
43	M	64	Seizures, cognitive dysfunction, anxiety and depression	Normal	Background: focal slow waves in bilateral frontal, central and temporal regions; Itcal:12 focal seizures originating from right frontal region	1:100/1:100	3	Corticosteroids+ IVIG+LEV+ Mycophenolate mofetil	Significantly effective	0
44	M	35	Weakness and hypoesthesia of bilateral lower extremities with pain	Normal	Normal	−/1:10	3	Corticosteroids+ IVIG	Significantly effective	0
45	F	52	Seizures, cognitive dysfunction, anxiety and depression	Normal	Background: focal slow waves in bilateral frontal, central and temporal regions; Interictal: sharps in right anterior temporal region	−/1:10	3	CBZ + Phenytoin+ Phenobarbital	Effective	2

CBZ, carbamazepine; CSF, cerebrospinal fluid; IVIG, intravenous immunoglobulin; LEV, levetiracetam; MRI, magnetic resonance imaging; OXC, oxcarbazepine; T2 FlAIR, T2 fluid attenuated inversion recovery; mRS, Modified Rankin Scale; NAA, N-acetyl aspartate.

##### 3.2.2.5 Anti-GAD65 encephalitis

**Figure 6:**
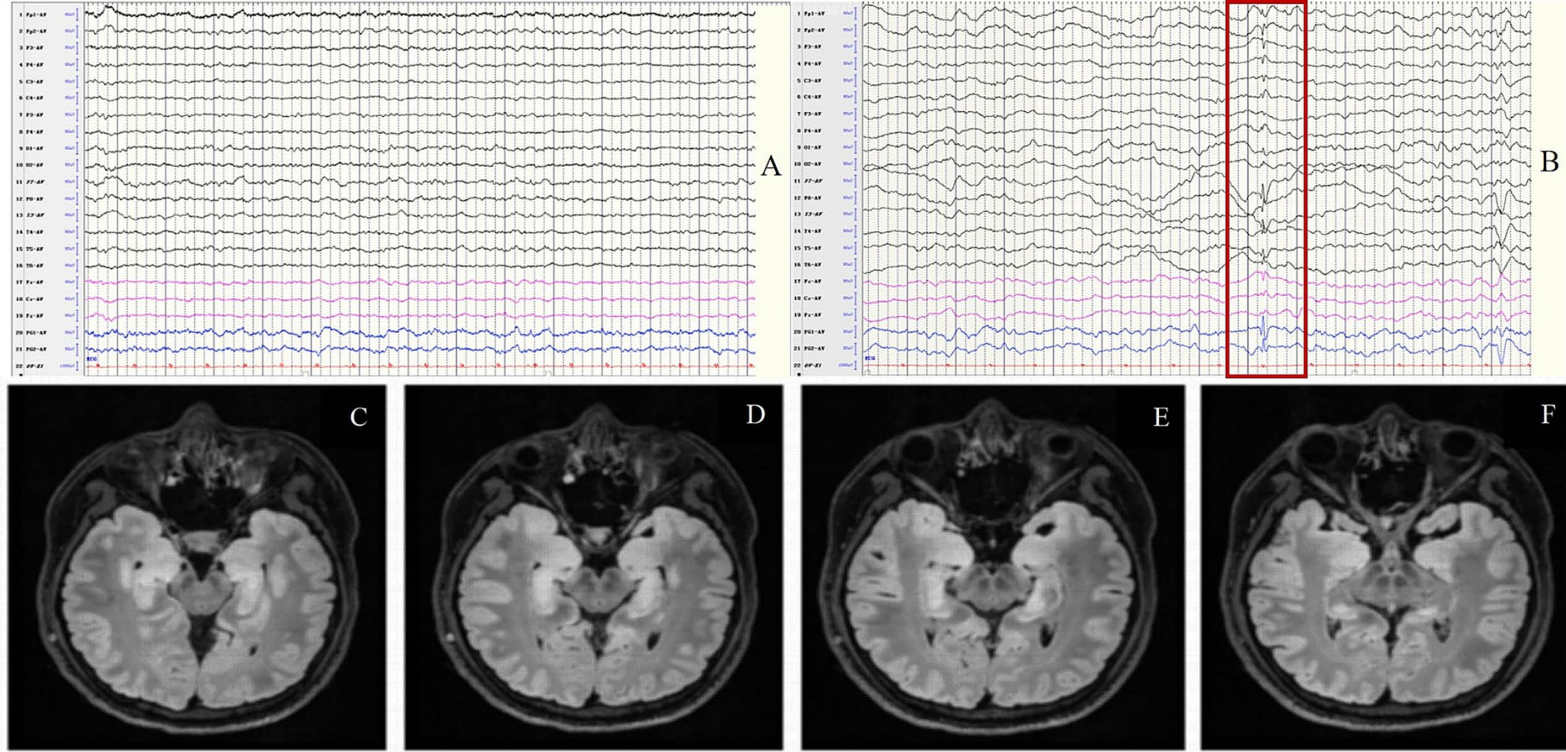
Case 51, female, 33 years old, who was diagnosed with anti-GAD65 encephalitis. **(A,B)** Fifteen days after onset and before immunotherapy, EEG showed focal slow waves in the background activity **(A)** and spikes in bilateral anterior temporal regions **(B)**; **(C–F)** Thirty days after onset and before immunotherapy, brain MRI showed swelling and T2 FLAIR hyperintensity in the bilateral hippocampus. The red border signifies the spikes in bilateral anterior temporal regions.

In this group, all 6 patients were females The average onset age was37 ± 8.99 years (range 26–52). The median disease duration was 547 days. EEG abnormalities were present in all patients (100%). The background activity was abnormal in 100% of cases, manifesting as focal slow waves mainly involving the unilateral (16.7%) or bilateral (83.3%) temporal regions (Figure 6); IEDs were observed in 5 patients (83.3%) mainly involving the temporal regions. No ictal EEG was detected.

Three of the 6 patients underwent 24-h vEEG 5 to 12 months after immunotherapy, among them 1 revealed no significant improvement with slowing of background activity and IEDs, 1 showed normal EEG 5 months after immunotherapy, and 1 revealed significant improvement with reduced slow waves and IEDs 6 months after immunotherapy. The clinical and EEG characteristics of anti-GAD65 encephalitis were shown in Table 5.

**Table 5:** Clinical and electroencephalographic characteristics of Anti-Glutamate Decarboxylase 65 (GAD65) Encephalitis.

Case	Sex	Age	Clinical manifestations	Brain MRI	vEEG	GAD65 Ab titer in CSF/serum	Disease severity (mRS)	Treatment	Therapeutic response	Outcome (mRS)
46	F	32	Cerebellar ataxia, hyperthyroidism	T2 FLAIR hyperintensity and diffusion restriction in right brachium pontine and left cerebellopontine crus	Background: focal slow waves in bilateral frontal, central and temporal regions	1:100/1:100	3	Corticosteroids+ IVIG	Significantly effective	0
47	F	52	Seizures, cognitive dysfunction, anxiety and depression, T1DM	T2 FLAIR hyperintensity and diffusion restriction in bilateral hippocampal	Background:focal slow waves in bilateral temporal regions; Interictal: spikes in left temporal regions	1:100/1:100	3	Corticosteroids+ IVIG+LEV	Ineffective	3
48	F	26	Seizures, anxiety and depression, T1DM	Normal	Background:focal slow waves in bilateral frontal, central and temporal regions; Interictal: sharps in left posterior temporal region	1:100 /1:100	2	Corticosteroids+ IVIG+LEV+LTG	Significantly effective	0
49	F	38	Seizures, anxiety, cognitive dysfunction, T1DM	Normal	Background: focal slow waves in left frontal, central and temporal regions; Interictal: sharps in bilateral frontal, central and temporal regions	1:32 /1:100	3	Corticosteroids+ IVIG+OXC+ LEV	Ineffective	3
50	F	41	Seizures, cognitive dysfunction, anxiety and depression, T1DM	Bilateral frontotemporal atrophy	Background: focal slow waves in bilateral frontotemporal regions; Interictal: spikes in bilateral temporal regions	1:100/1:100	3	Corticosteroids+ LEV	Significantly effective	0
51	F	33	Seizures, mental and behavioral abnormalities, cognitive dysfunction, hyperthyroidism	Swelling and T2 FLAIR hyperintensity in bilateral hippocampus	Background: focal slow waves in bilateral anterior temporal regions; Interictal: spikes in bilateral anterior temporal regions	1:320/1:320	5	Corticosteroids+ IVIG+ LTG+VPA	Significantly effective	1

CSF, cerebrospinal fluid; IVIG, intravenous immunoglobulin; LEV, levetiracetam; LTG, lamotrigine; MRI, magnetic resonance imaging; OXC, oxcarbazepine; T1DM, type 1 diabetes mellitus; T2 FlAIR, T2 fluid attenuated inversion recovery; VPA, Valproate; mRS, Modified Rankin Scale.

##### 3.2.2.6 MOG antibody cortical encephalitis (FLAMES)

This group included 7 patients (4 males and 3 females) with a sex ratio of 4:3. The average onset age was 24.71 ± 12.65 years (range 14–49). The median disease duration was 21 days. EEG abnormalities were present in 100% of patients. The background activity was abnormal in all 7 patients (100%), showing diffuse slow waves in the bilateral (1/7) or unilateral (1/7) hemisphere in 2 patients (2/7) and focal slow waves in the unilateral hemisphere in 4 patients (4/7), involving the frontal (1/7), temporal (4/7), and occipital regions (1/7); 1 patient had focal slow waves in the bilateral frontal, central, and temporal regions. IEDs were detected in 5 patients (71.4%), presenting as spikes or sharps in the unilateral hemisphere in 4 patients (4/7) and PLEDs in 1 patient (1/7). One patient (1/7) had 20 focal seizures originating from the right central region.

**Figure 7:**
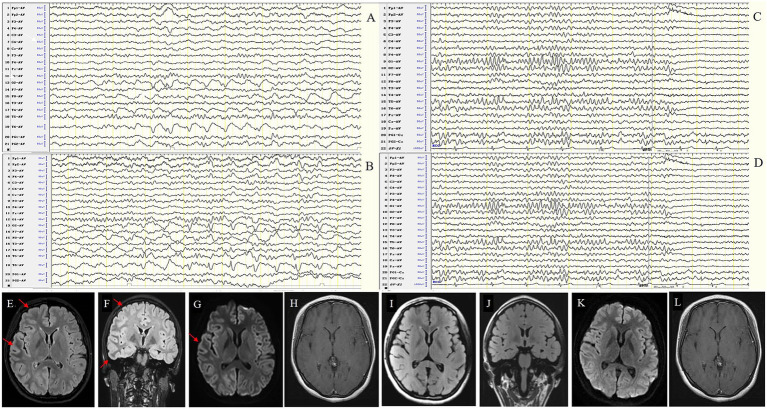
Case 54, female, 16 years old, who was diagnosed with MOG antibody cortical encephalitis (FLAMES). **(A,B)** Twenty-one days after onset and before immunotherapy, EEG showed diffuse slow waves in the right hemisphere in background activity **(A)** and interictal epileptic discharges in the right occipital and posterior temporal regions **(B)**. MRI shows swelling of the left frontotemporal cortex **(E–H)**. **(C,D)** Ninety days after immunotherapy, EEG showed significant improvement in background activity without interictal epileptic discharges. MRI showed that the swelling of the left frontotemporal cortex was improved **(I–L)**.

Three of the 7 patients underwent 24-h vEEG 10–18 months after immunotherapy. All experienced clinical remission, one showed normal EEG (Figure 7), one still had slow waves in the left hemisphere, and another exhibited significant improvement in background activity but relapsed 1 year later with optic neuritis due to irregular drug withdrawal. The clinical and EEG characteristics of MOG antibody cortical encephalitis were shown in Table 6.

**Table 6:** Clinical and electroencephalographic characteristics of Myelin Oligodendrocyte Glycoprotein (MOG) Antibody Cortical Encephalitis.

Case	Sex	Age	Clinical manifestations	Brain MRI	vEEG	MOG Ab titer in CSF/serum	Disease severity (mRS)	Treatment	Therapeutic response	Outcome (mRS)
52	F	14	Seizures, headache, mental and behavioral abnormalities	Cortical swelling and T2 FLAIR hyperintensity in the left frontal and insular cortex, decreased NAA peak and increased Cho peak	Background: diffuse slow waves in left hemisphere; Interictal: periodic epileptic discharges in left hemisphere	1:1/1:32	5	Corticosteroids+ IVIG+LEV+ VPA	Significantly effective	2
53	M	16	Seizures, cognitive dysfunction, anxiety and depression	Cortical swelling and T2 FLAIR hyperintensity in right frontal cortex	Background:focal slow waves in bilateral frontal regions; interictal:spikes in right frontal regions	–/1:32	2	Corticosteroids+ LEV	Significantly effective	0
54	F	16	Seizures, headache, cognitive dysfunction, anxiety and depression	Cortical swelling and T2 FLAIR hyperintensity in right hemisphere, with diffusion restriction	Background: diffuse slow waves in right hemisphere; Interictal: sharp and wave complexes in right occipital and temporal regions	1:10/1:32	4	Corticosteroids+ LEV	Significantly effective	2
55	M	33	Seizures, cognitive dysfunction, anxiety and depression	Cortical swelling and T2 FLAIR hyperintensity in bilateral frontal lobe and cingulate gyrus, with diffusion restriction, decreased NAA peak and increased Cho peak	Background: focal slow waves in right central and temporal regions; Interictal: sharps in right central and parietal regions	–/1:32	3	Corticosteroids+ IVIG+ OXC	Effective	2
56	M	26	Seizures, cognitive dysfunction, anxiety and depression	Atrophy and T2 FLAIR hyperintensity in right hippocampus	Background: focal slow wave in right temporal region; Interictal: sharps in right temporal region	–/1:10	4	Corticosteroids+VPA+ OXC+ LCM	Significantly effective	1
57	F	19	Seizures, cognitive dysfunction, mental and behavioral abnormalities	T2 FLAIR hyperintensity in left thalamus and right frontal and parietal regions	Background: Delta activity in right anterior temporal region; Interictal: spikes in right anterior temporal region and bilateral occipital regions	1:10/1:10	4	Corticosteroids+IVIG+ LEV	Significantly effective	0
58	M	49	Seizures, cognitive dysfunction, anxiety and depression	T2 FLAIR hyperintensity in right frontal, temporo-occipital junction	Background: focal slow waves in bilateral frontal, central, and temporal regions; Ictal: 20 focal seizures originating from right central region	1:10/1:10	5	Corticosteroids+IVIG+ LEV+ CBZ	Effective	2

CBZ, carbamazepine; CSF, cerebrospinal fluid; IVIG, intravenous immunoglobulin; LEV, levetiracetam; LCM, Lacoxamide; MRI, magnetic resonance imaging; OXC, oxcarbazepine; T2 FlAIR, T2 fluid attenuated inversion recovery; TPM, topiramate; VPA, Valproate; mRS, Modified Rankin Scale; NAA, N-acetyl aspartate; Cho, choline.

##### 3.2.2.7 GFAP-A

In this group, both patients were males, 69 and 70 years old, respectively. The disease duration was 6 days and 8 days, respectively. EEG abnormalities were present in both patients. One showed abnormal background activity with bilateral frontotemporal delta activity, the other revealed diffuse slow waves accompanied by triphasic waves in bilateral frontal regions and PLEDs, and 2 focal seizures with impaired consciousness (FIAS) originating from left frontal region were detected.

One of the 2 patients underwent 24-h vEEG 6 months after immunotherapy, showing significant improvement with reduced slow waves and IEDs, no ictal EEG was detected. The clinical and EEG characteristics of GFAP-A encephalitis were shown in Table 7.

**Table 7:** Clinical and electroencephalographic characteristics of Glial fibrillary acidic protein astrocytopathy (GFAP-A).

Case	Sex	Age	Clinical manifestations	Brain MRI	vEEG	GFAP-A Ab titer in CSF/serum	Disease severity (mRS)	Treatment	Therapeutic response	Outcome (mRS)
59	M	70	Cognitive dysfunction	Normal	Background: delta activity in bilateral frontotemporal regions	1:32/−	3	Corticosteroids	Effective	2
60	M	69	Seizures, cognitive dysfunction, anxiety and depression	T2 FLAIR hyperintensity in bilateral frontotemporal lobes	Background: diffuse slow waves; Interictal: paroxysmal triphasic waves in bilateral frontal regions; Ictal: 2 focal impaired awareness seizures originating from right frontal region	1:100/1:100	5	Corticosteroids+CBZ+ LEV+VPA	Significantly effective	1

CBZ, carbamazepine; CSF, cerebrospinal fluid; IVIG, intravenous immunoglobulin; LEV, levetiracetam; MRI, magnetic resonance imaging; T2 FlAIR, T2 fluid attenuated inversion recovery; VPA, Valproate; mRS, Modified Rankin Scale.

### 3.3 Correlation analysis between EEG severity grading and clinical data

#### 3.3.1 Correlation analysis

**Figure 8:**
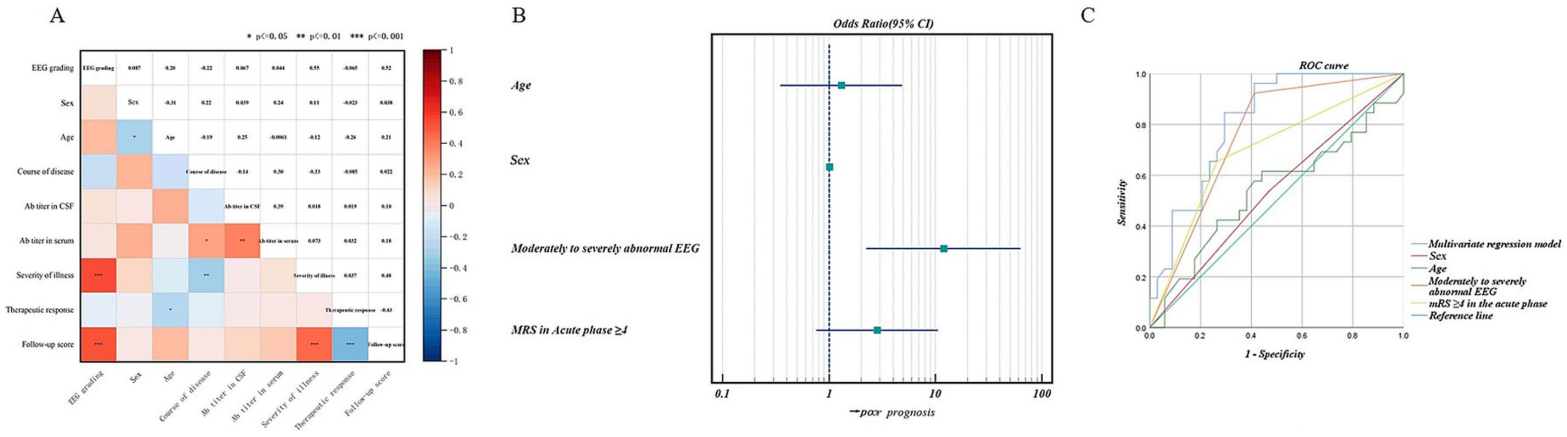
**(A)** Spearman correlation coefficient heatmap between EEG severity grading and serum or cerebrospinal fluid antibody titers, age, sex, course of disease, disease severity, treatment response, and prognostic score of antibody-mediated AE. The asterisks (*) denote the statistical significance of the Spearman correlation coefficients, with * indicating *p* < 0.05, ** indicating *p* < 0.01, and *** indicating *p* < 0.001. **(B)** Forest plot of binary logistic regression. The presence of moderate to severe abnormal EEG was a significant predictor of poor prognosis. **(C)** ROC curves for predicting prognosis using univariate and multivariate analyses. ROC curves analysis showed that moderate to severe abnormal EEG had the highest predictive value for prognosis, with an AUC (the area under the curve) of 0.756 (0.632–0.879), improving to 0.812 (0.705–0.919) with multivariate analysis.

Fifty-eight out of 60 AE patients showed abnormal EEG during the acute phase, including 6 with borderline, 14 with mildly abnormal, 17 with moderately abnormal, 20 with severely abnormal, and 1 with extremely severely abnormal EEG. Patients with normal, borderline, or mildly abnormal EEG had good prognosis in 90.9% of cases, while patients with moderately, severely, or extremely severely abnormal EEG had good prognosis in 31.6% of cases and poor prognosis in 68.4% of cases. Correlation analysis was performed between EEG severity grading and antibody titers, disease severity, treatment response, and prognosis score. The results indicated that EEG severity grading positively correlated with disease severity (*r* = 0.547, *p* < 0.0001) and prognosis score (*r* = 0.521, *p* < 0.0001), but not correlated with antibody titers and treatment response (*p* > 0.05). Figure 8A showed the spearman correlation coefficient heatmap.

#### 3.3.2 Binary logistic regression and ROC curve analysis

To further assess whether EEG severity grading is an important factor in predicting prognosis, patients were categorized into two groups based on their prognostic scores: a good prognosis group with a score ≤ 1 (assigned as 0) and a poor prognosis group with a score ≥ 2 (assigned as 1). Univariate logistic regression screening showed that moderate to severe abnormal EEG (OR = 17.143, *p* < 0.05) and mRS ≥4 in the acute phase (OR = 5.247, *p* < 0.05) were possible risk factors for poor prognosis. Binary logistic regression analysis was conducted with age and gender as correction factors, and we found that moderate to severe abnormal EEG was a significant predictor of poor prognosis (OR = 11.942, *p* < 0.05). Figure 8B showed the binary logistic regression forest plot.

ROC curve analysis (Supplementary Table S3; Figure 8C) showed that moderate to severe abnormal EEG had the highest predictive value for prognosis, with an AUC (the area under the curve) of 0.756 (0.632–0.879), improving to 0.812 (0.705–0.919), with multivariate analysis.

## 4 Discussion

EEG plays a crucial role on the diagnosis of antibody-mediated AE. This study analyzed and post-processed the long-term vEEG data of 60 AE patients to identify common and subtype-specific EEG characteristics, as well as their role on diagnosis, treatment, and prognosis. The key findings are as follows: (1) High Sensitivity of EEG: EEG abnormalities were present in 96.7% of patients during the acute phase, higher than imaging abnormalities (66.7%). (2) Common EEG Patterns: The acute phase was marked by focal or diffuse slow-wave activity, IEDs in temporal or extratemporal regions, and frequent clinical and subclinical seizures. Significant improvements in EEG were observed during the recovery phase. (3) Subtype-Specific EEG Patterns: Different subtypes of AE exhibited distinct EEG characteristics. Anti-LGI1 encephalitis presented two primary clinical-electroencephalographic patterns: one was MTLE-like seizure with ictal activity originating from the temporal region; the other was FBDS with ictal EEG showing generalized electro-decremental activity before or at the onset of seizure with extensive infra-slow activity. Anti-NMDAR encephalitis was marked by abnormal background activity, including extreme delta brush, frontotemporal delta activity, diffuse or focal slow waves, with scattered and unfixed IEDs. MOG antibody cortical encephalitis usually presented as diffuse or focal slow waves in unilateral hemisphere accompanied by ipsilateral IEDs, sometimes with PLEDs. Anti-GABA_B_R and anti-GAD65 encephalitis usually manifested as slow waves, IEDs and ictal activity involving the temporal regions. (4) Prognostic Value of EEG: The EEG severity grading positively correlated with disease severity and prognosis score, moderate to severe abnormal EEG was a risk factor for poor prognosis.

### 4.1 Common EEG features of antibody-mediated AE

Previous studies have shown that around 85% of AE patients exhibit EEG abnormalities, primarily slower background activity (51.1%), followed by IEDs (21.6%), with a higher prevalence in the temporal than frontal lobe. Focal seizures and subclinical seizures were frequent (69.6%), originating from various regions including the frontal, temporal, central, and parietal regions (9, 10). Baysal-Kirac et al. reported that 71% of AE patients had persistent delta or theta rhythm, 40% exhibited prefrontal lobe discontinuous delta rhythm (FIRDA); in contrast, only 25% of epilepsy or encephalopathy patients had persistent delta or theta rhythm, and 5% had FIRDA (14). This study found a higher rate of EEG abnormalities (96.7%), emphasizing the superior sensitivity of EEG over imaging for detecting AE-related brain dysfunction. Most cases exhibited focal (86.7%) or diffuse (10%) slow waves, and 30% showed delta rhythm. IEDs were detected in 61.7% of patients, with clinical seizures in 31.7% and subclinical seizures in 5% of patients. These were consistent with previous studies (15, 16). We also found that the EEG patterns changed along with the course of disease and immunotherapy. During the recovery phase, 92.6% of 27 AE patients who underwent long-term vEEG after immunothery showed significant improvement in EEG, with reduced slow waves and IEDs, indicating that EEG is a sensitive auxiliary examination for AE that can assis not only early identification and diagnosis, but also evaluation of disease sevirity and treatment response. These findings need to be verified in the fututure study with a larger sample size and extended follow-up period.

### 4.2 EEG biomarkers of different antibody-mediated AEs

#### 4.2.1 Anti-NMDAR encephalitis

Anti-NMDAR encephalitis is the most prevalent form of AE, constituting approximately 70% of cases (17). As shown by previous studies, the EEG abnormal rate of anti-NMDAR encephalitis ranged from 80 to 100%, common EEG abnormalities included: slowing or disappearance of background activity, diffuse or focal slow waves, superimposed IEDs, as well as frequent focal or subclinical seizures, and some patients presented with status epilepticus (18). Focal seizures have been reported to originate from the temporal lobe or surrounding brain areas (19, 20). Several EEG patterns have been suggested as potential biomarkers for anti-NMDAR encephalitis, including EDB, beta:delta ratio (BDR), extreme beta activity (EBA), and generalized rhythmic delta activity (GRDA) (21). EDB, first described by Schmitt et al. (22), was seen in 16–41% of patients (23, 24) and associated with severe cases, prolonged disease duration and poor prognosis (25). In this study, only one case exhibited EDB, who still had seizures half a year after immunotherapy, indicating poor prognosis. Jeannin-Mayer et al. (26) performed EEG analysis for 24 patients with anti-NMDAR encephalitis and found that EBA was present in 71%, EDB in 58%, and GRDA in 50% of patients. Schmit et al. (22) showed that all cases had diffuse slowing of background activity, particularly in the frontotemporal regions, and more than half of the cases had GRDA, and 25–50% had EBA, but the influence of sedative drugs could not be excluded.

In this study, 91.7% of patients showed EEG abnormalities, mainly manifesting as abnormal background activity, including EBD, frontotemporal delta activity or rhythm, diffuse or focal slow waves. Only one case exhibited EDB, possibly due to milder disease in this cohort. IEDs were detected in 58.3% of patients with location scattered and unfixed, which was specific and different from AE mediated by other antibodies, reflecting the diffuse cortical involvement.

#### 4.2.2 Anti-LGI1 encephalitis

Anti-LGI1 encephalitis is the second common type of AE (27, 28). Previous studies by our group and other scholars showed that patients with anti-LGI1 encephalitis can be subdivided into following 3 groups based on seizure semiology: MTLE-like seizure, FBDS and MTLE-like seizure + FBDS group (8, 29). The clinical presentation, imaging and EEG features were different between different subgroups. In this study, we performed subgroup analysis for EEG of anti-LGI1 encephalitis. In the MTLE-like seizure group, slow waves in the background and IEDs involved the temporal regions, ictal EEG showing the temporal region origin, which was consistent with imaging that usually affected the hippocampus or medial temporal lobe. In the FBDS group, the location of focal slow waves and IEDs were unfixed, ictal EEG showing generalized electro-decremental activity before or at the onset of seizure with extensive infra-slow activity superimposed with EMG artifacts, indicating deeply located and highly localized epileptogenic zone, which was consistent with imaging that usually affected subcortical basal ganglia. In the MTLE-like seizure + FBDS group, both EEG patterns were present. These data indicated distinct EEG features of different subgroups of anti-LGI1 encephalitis, which can help accurate diagnosis and treatment.

Previous studies showed that the abnormal rate of ictal EEG of FBDS was only 13–40% (30, 31), presenting as electrodecremental activity (32) or only EMG artifacts (31). Recent studies revealed an electrodecremental pattern and frontal slow or infraslow activity preceding FBDS, which may be a characteristic ictal EEG avtivity of FBDS (27, 33, 34). Our study confirmed these findings and showed that the ictal EEG of FBDS included 3 components, generalized electrodecremental activity before or at the onset of seizure, extensive multiphase infraslow waves (frequency < 0.5 Hz), and superimposed EMG artifacts. We further analyzed the parameters of ictal EEG of 73 times FBDS, and found that 67 times were preictal electrodecremental activity (preictal 0.6 s–4.9 s), 6 times were electrodecremental activity at the onset of seizures; all the 73 episodes showed infraslow waves with amplitude ranging from 68.4 to 1,000 uV, frequency ranging from 0.2 to 0.7 Hz, and duration ranging from 0.8 to 2.8 s. EMG were simultaneously recorded in 44 episodes and the EEG changes appeared 0 s-2.8 s before EMG changes. These data were helpful for further identification of ictal EEG pattern of FBDS. We speculated that “generalized electrodecremental activity before or at the onset of seizures with extensive infraslow waves superimposed with EMG artifacts” was a specific EEG biomarker for FBDS, prompting doctors to detect LGI1 antibodies as soon as possible so as to shorten the diagnosis and treatment period.

#### 4.2.3 Anti-GABA_B_R encephalitis

Anti-GABA_B_R encephalitis was first reported by Lancaster et al. (3), which is characterized by early-onset seizures and limbic encephalitis, commonly associated with tumors, especially small-cell lung cancer. According to previous studies, EEG abnormalities were found in 86% of patients with anti-GABA_B_R encephalitis (35), 50.5% of patients showed diffuse or focal slow waves, 38.1% exhibited temporal IEDs, ictal EEG suggested medial temporal lobe origin. Normal EEG has also been reported and may be a predictor for good prognosis (36, 37). A recent report revealed that the distribution of slow wave activities reflected disease severity and may be related to disease recurrence (38).

Similar to previous studies, our study showed that the abnormal rate of EEG in the acute phase of anti-GABA_B_R encephalitis was 100%, manifesting as temporal slow waves and IEDs, with subclinical seizures originating from the temporal region. These findings were consistent with the imaging, which often showed T2 FLAIR hyperintensity in the temporal lobe or hippocampus. However, whether there are specific EEG features in anti-GABA_B_R encephalitis compared with other limbic encephalitis, remains unclear and need further investigation with larger sample size and longer follow-up period.

#### 4.2.4 Anti-Caspr2 encephalitis

Anti-Caspr2 encephalitis, though variable in its clinical presentations, lacks a specific EEG signature. Previous studies reported an abnormal EEG rate of approximately 65%, showing slower background activity, focal (14%) or diffuse (30%) slow waves, IEDs (47%), and unilateral or bilateral hemispheric spike and wave complexes (39–42).

In this study, 75% of patients had abnormal EEG, with focal slow waves in the frontal, central, and temporal regions, IEDs involving the temporal regions. Ictal EEG were detected in 2 patients (50%), one originating from right frontal region, the other with unclear origin. These results are a little different from previous studies. Further investigation with larger sample size and extended follow-up period is required to clarify EEG characteristics of this subtype.

#### 4.2.5 Anti-GAD65 encephalitis

GAD antibodies were first described in 1988 and the first confirmed synapse protein antibodies, which exist in many autoimmune neurological diseases, including stiff person syndrome, temporal lobe epilepsy, cerebellar ataxia, limbic encephalitis, these syndromes constitute the “GAD antibody spectrum disorders” (GAD-SDs) (43). EEG plays an important role on the epilepsy and limbic encephalitis phenotype of GAD-SDs. As shown by previous studies, the EEG changes in GAD-SDs were non-specific, approximately 66% showed ictal or interictal epileptiform discharges in the temporal regions, some with slowing of background activity, and some showed normal EEG in the early stage of disease (16, 44).

In our study, 100% of patients exhibited abnormal EEG, mainly slow waves and IEDs in the temporal regions, suggesting involvement of the limbic system, particularly the hippocampus and mesial temporal lobe. Some patients showed diverse focal abnormal electrical activities which were not limited to the temporal lobe, may be related to widespread cortical damage and the establishment of abnormal neural network and circuits.

#### 4.2.6 MOG antibody cortical encephalitis

MOGAD involve various locations of the central nervous system, with cortical encephalitis being a less common presentation. Recently the acronym “FLAMES” (“FLAIR-hyperintense Lesions in Anti-MOG-associated Encephalitis with Seizures”) has been proposed to describe a special clinico-radiological syndrome of MOGAD, which manifest as seizures, headache, fever, unilateral or bilateral cortical swelling and T2 FLAIR hyperintensity on the MRI (45). To date, the clinical and EEG features of FLAMES remain unclear. Foiadelli et al. (46) performed a literature review and analyzed the EEG of 30 patients with MOGAD presenting seizures, and found that common EEG abnormalities included focal (56.7%) or diffuse (33.3%) slow waves, with or without IEDs. Ramanathan et al. (47) provided an ictal EEG of a 3-year-old female with MOGAD, showing focal slow waves accompanied by spikes, with tonic gaze and jerks of right face and hand. Recent review by Wang et al. showed the abnormal EEG of FLAMES presented non-specific slow waves in the background consistent with the cortical lesion location, IEDs and other abnormalities (48, 49).

In this study, 100% of patients with MOG antibody cortical encephalitis had EEG abnormalities, including diffuse or focal slow waves in unilateral or bilateral hemisphere accompanied by ipsilateral IEDs, sometimes with PLEDs, often correlating with lesion locations on brain MRI. EEG improvements were noted after immunotherapy, indicating its potential in evaluating treatment response.

#### 4.2.7 GFAP-A

GFAP-A is a novel autoimmune disease affecting the nervous system that was first defined in 2016 (7). There were few reports focusing on EEG abnormalities of GFAP-A. A previous study (50) revealed non-specific EEG changes, including diffuse slow waves and IEDs (19.23%). Theroux et al. (51) reported a child with GFAP-A whose EEG showed EDB.

In this study, both patients had EEG abnormalities, one showed bilateral frontal and temporal delta activity in the background; the other revealed diffuse slow waves with triphasic waves in bilateral frontal regions and unilateral periodic discharges, and 2 FIAS originating from left frontal region, which were specific and different from previous studies. However, no characteristic EEG pattern can be found due to small sample size. In the future, we will expand the sample size and prolong EEG recording and follow-up period to further clarify the EEG features of GFAP-A.

### 4.3 EEG as a prognostic biomarker in antibody-mediated AE

EEG has been shown to be a valuable tool for the diagnosis, therapy response and prognosis assessment in antibody-mediated AE. Several previous studies have linked more severe EEG abnormalities to worse outcomes, particularly in cases with status epilepticus or refractory seizures. Recent studies showed that lateral periodic or rhythmic discharges (PLEDs), epileptic seizures, and new-onset refractory status epilepticus increased the risk of poor prognosis, regardless of AE subtypes (15, 52). The correlation between EDB and prognosis was controversial. Several studies reported that EDB was associated with poor prognosis (20, 22, 25), while others displayed no association between EDB and prognosis (24, 26, 53). To date, there is still lack of studies on EEG predicting the prognosis, and the relationship between EEG severity grading and disease severity, antibody titer, treatment response, and prognosis remains unknown. In this study, the severity of EEG changes in the acute phase of AE positively correlated with both disease severity and prognosis scores. Patients with moderate to severe EEG abnormalities were more likely to have poor outcomes, highlighting the potential of EEG as a sensitive and specific biomarker for prognosis of AE. However, due to limitations in sample size and follow-up period in this study, additional statistical methods and prediction model were not employed at this time. In the future, we plan to expand the sample size and extend follow-up period, as well as collaborating with experts in science and engineering to implement quantitative EEG post-processing technologies and advanced statistical methodologies, thereby developing a more accurate EEG prediction model to assist in the precise diagnosis and treatment of AE.

## 5 Conclusion

EEG abnormalities are more prevalent than imaging abnormalities in the acute phase of antibody-mediated AE, and exhibit common and specific features across different subtypes. The EEG severity grading positively correlates with disease severity and prognosis score, and moderate to severe abnormal EEG is a risk factor for poor prognosis, suggesting that EEG is a sensitive and specific tool for antibody-mediated AE. Further investigation with a larger sample size and a longer follow-up period is required, utilizing quantitative EEG post-processing technologies and advanced statistical methodologies, to develop a more accurate EEG prediction model for the precise diagnosis and treatment of AE.

## Data Availability

The original contributions presented in the study are included in the article/Supplementary material, further inquiries can be directed to the corresponding authors.

## Supplementary material

Figure S1 Sex distribution map of different subtypes of antibody-mediated AE.

Figure S2 Distribution map of abnormal EEG findings across different subtypes of antibody-mediated encephalitis.
