# Supplementary figures and images for: Electroencephalographic biomarkers of antibody-mediated autoimmune encephalitis
Source: Front Neurol. 2025 Mar 26;16:1510722. doi: 10.3389/fneur.2025.1510722 (PMC11981172; doi:10.3389/fneur.2025.1510722)

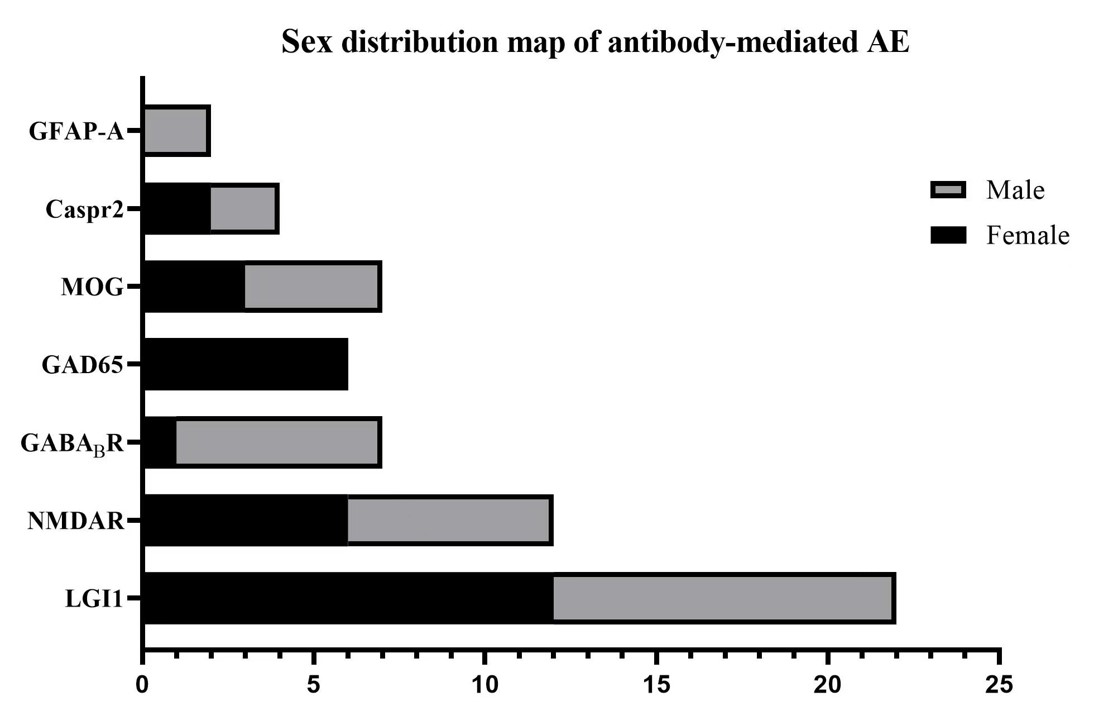

Supplement: Supplementary file 1 [file Data_Sheet_1.ZIP › Supplementary Figure 1.jpg]

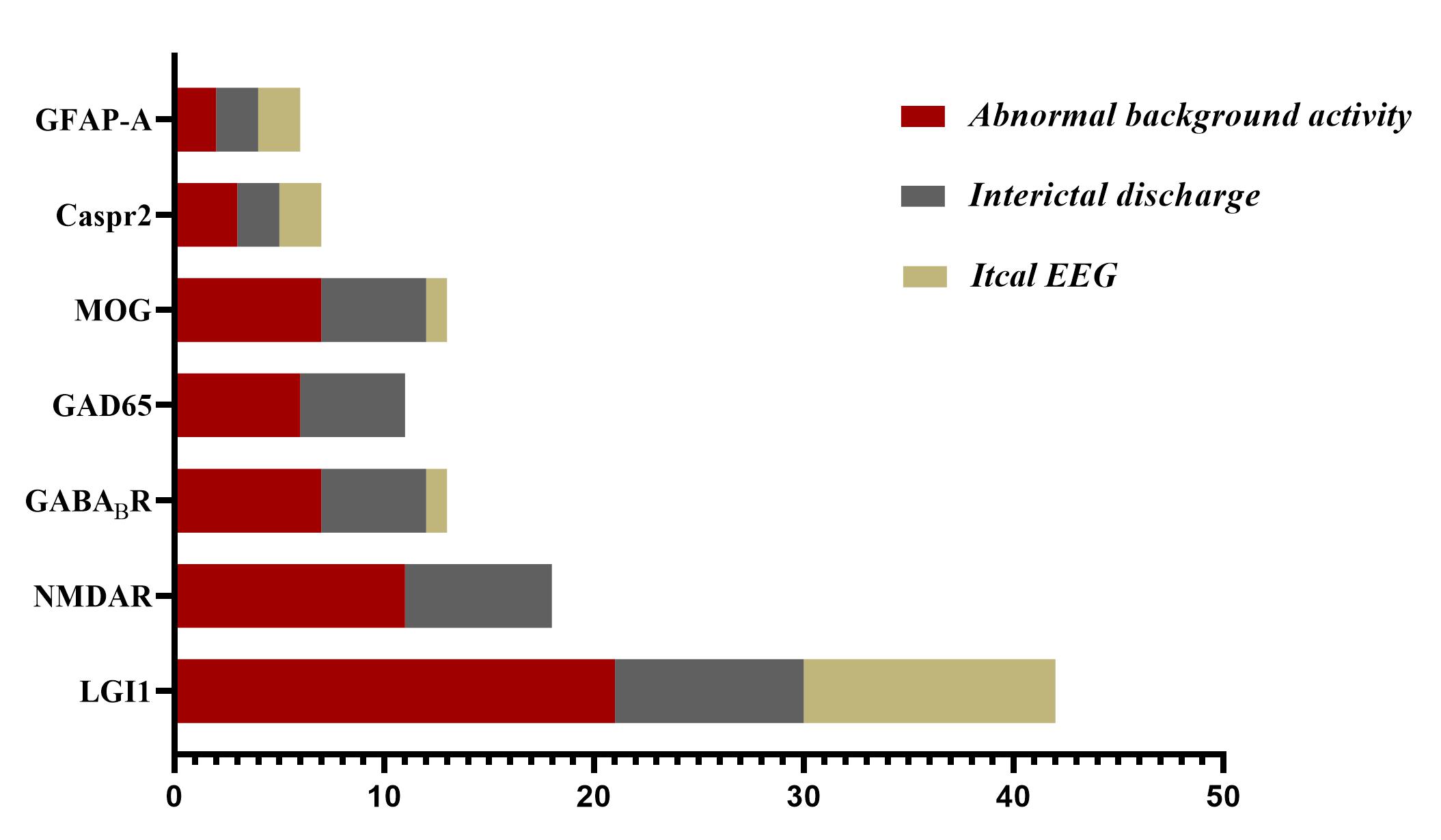

Supplement: Supplementary file 1 [file Data_Sheet_1.ZIP › Supplementary Figure 2.jpg]
